# Spatial representations of the viewer’s surroundings

**DOI:** 10.1038/s41598-018-25433-5

**Published:** 2018-05-08

**Authors:** Satoshi Shioiri, Masayuki Kobayashi, Kazumichi Matsumiya, Ichiro Kuriki

**Affiliations:** 1 Research Institute of Electrical Communication, Tohoku University, Sendai, Japan; 2 Graduate School of Information Sciences, Tohoku University, Sendai, Japan

## Abstract

Spatial representation surrounding a viewer including outside the visual field is crucial for moving around the three-dimensional world. To obtain such spatial representations, we predict that there is a learning process that integrates visual inputs from different viewpoints covering all the 360° visual angles. We report here the learning effect of the spatial layouts on six displays arranged to surround the viewer, showing shortening of visual search time on surrounding layouts that are repeatedly used (contextual cueing effect). The learning effect is found even in the time to reach the display with the target as well as the time to reach the target within the target display, which indicates that there is an implicit learning effect on spatial configurations of stimulus elements across displays. Since, furthermore, the learning effect is found between layouts and the target presented on displays located even 120° apart, this effect should be based on the representation that covers visual information far outside the visual field.

## Introduction

Vision for action has been one of the most important topics in vision science for decades^1–3^. The influence of the body on vision has been shown, as in visual facilitation near body^4–7^, suggesting specific processing in the space near the body (peripersonal space, PPS^8,9^). To movie in the three-dimensional (3D) world, recognition of the surrounding environment is crucial for vision for action. A representation of the surrounding environment is necessary for smooth movements and efficient actions. It has been demonstrated that mental models or representations of scenes around the viewer can be constructed with both visual and nonvisual information^10^. If we focus on vision for action, the PPS is the representation that is most relevant among several spatial representations^11,12^. The physiological finding of monkey neurons with specifically sensitive to stimuli presented near the body support the idea of PPS, as well as the finding of a greater neural response near one’s hand in human brain imaging^13^.

To construct the PPS or other types of spatial representation around the viewer, it is not sufficient to process retinal information obtained from a certain viewpoint at a certain moment. Rather, integrating information across saccadic eye movements is necessary. Several lines of research support integration across saccades although exactly what is integrated remains an unanswered question^14–16^. Context influence is another factor that should be considered in order to understand the constructing process of spatial representations^10,17,18^. One of examples of such contextual effects is shown in a boundary extension error in memory recalling^10,19^. The boundary extension is a phenomenon, where people remember seeing a surrounding region of a scene that was not visible in the view presented for memorization. The memorized representation of a scene does not simply depend on the current visual information, but also on contextual information. Contextual information likely extends scene representations, with the background, being extrapolated to neighboring regions.

To build the representation of 3D space surrounding the viewer, the visual system must combine visual information obtained by not only from different viewpoints but also from different viewing angles, moving the head and body as well as the eyes to scan invisible fields beyond the limit of the visual field at a fixation point. Daily experience suggests that people have a sense of locations for objects in their surroundings, and that there exist some kinds of representations of the spatial information around the viewer, including the area to the person’s rear. One can direct a hand toward a can of beer on a table behind their body without looking it, and then perhaps glance at the can before grabbing it for precise control. This kind of action was shown to be accompanied by large saccadic eye movements directed beyond the limit of the visual field while performing everyday tasks, such as tea-making^20^.

Studies of spatial memory have investigated the organization of the memorized spatial representations. Some studies have shown that a small number of cues such as landmark locations act as references to the environment^21–23^ while others have demonstrated contributions of 3D scene representations obtained visually^24,25^. Since scene representations can be learned implicitly whereas landmarks should be explicit, there are likely different underlying mechanisms for these two phenomena. One is a vision-based process that builds scene representation, and the other is a high-level process related to language that uses symbolic maps (i.e., conceptual representation). Our interest is in the visual process, which is likely used to control action more directly and without conscious effort in comparison with conceptual representation. Several studies have suggested that the visual system employs scene representations that are unlike photographs or 2D images. Influence of depth has been reported for the memory of spatial layouts^24,26^ and viewpoint invariance has also been demonstrated^24,27^. These studies of scene representation are limited, however, in terms of spatial extent to several tens of degrees^24,28,29^. For smooth action in the 3D space, information about the scene around the viewer is perhaps indispensable, and we expect that the visual system uses scene representations of the surrounding areas, including those outside the visual field.

**Figure 1:**
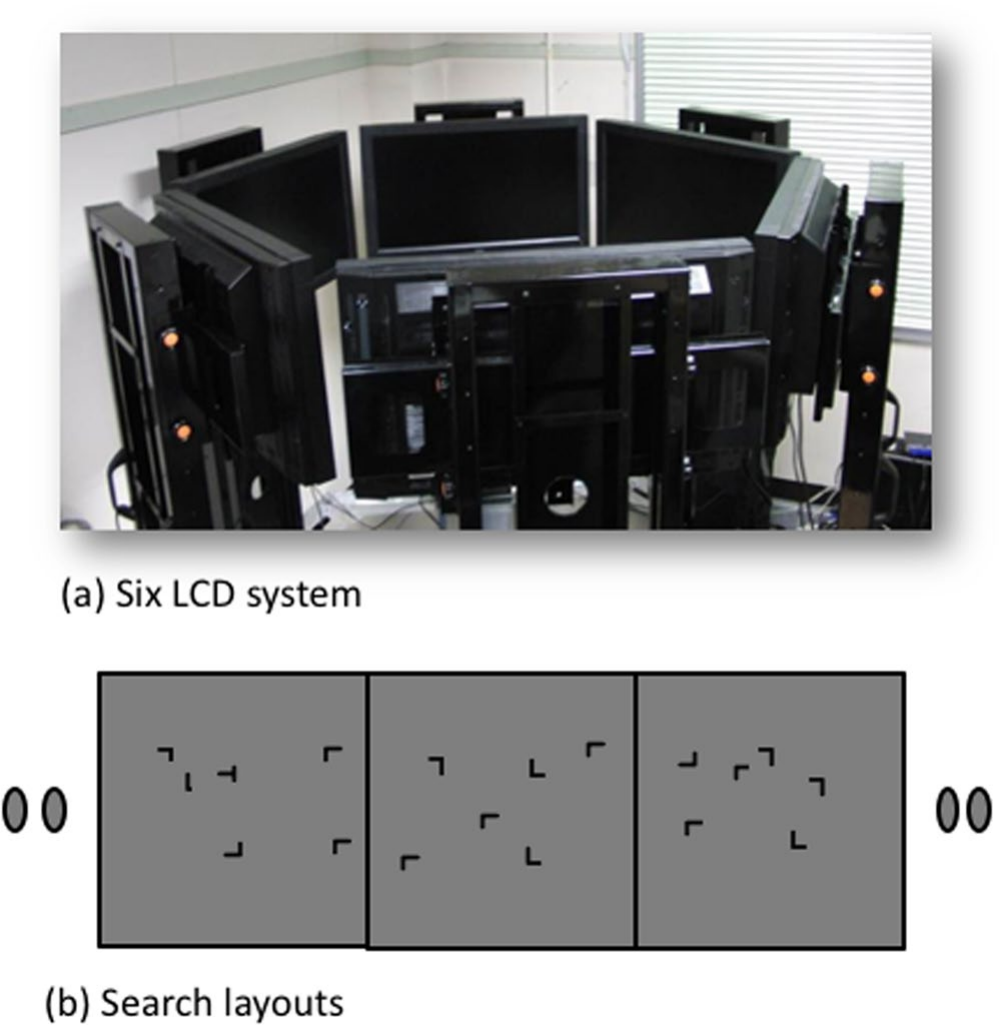
(**a**) Experimental setup. Six LCDs were arranged to surround the observer. One target (“T”) and 35 distractors (“L”) were presented on the six displays. (**b**) Stimulus display. Stimulus letters were arranged randomly with restriction that the number of the letters on each display was kept constant as six (six Ls or five Ls with a T). The locations of items were chosen randomly from intersections of an invisible 12 × 8 grid on each display.

In the present study, we investigated the scene representation of the surrounding, or 360° view. Implicit visual process was in focus to avoid the influence of conceptual processes. Our purpose was to investigate representations used for moving around familiar locations in the everyday life without conscious effort for accessing conceptual representations. It might be the case that implicit processes control the daily actions in familiar places in general. In order to isolate implicit processes from conceptual processes, we used the contextual cueing effect (CCE), which is a learning effect of the spatial layout in visual search displays that is known to be implicit^30^. In the experiment, observers repeatedly searched for a target among distractors in a visual search task. Half of the display layouts (i.e., the spatial distributions of the target and distractors within the displays) were repeatedly used throughout the experiment, whereas the other half were unique to each trial. The CCE makes target detection faster in the repeated layouts without awareness of the repetition. The CCE has been shown for layouts with depth information or 3D layouts^24,26^ which suggest that the underlying mechanism of the CCE represents 3D scenes. If we find the CCE for 360° layouts surrounding the viewer, this would indicate that there are representations for objects and space surrounding the viewer, integrating visual information from different visual directions. We conducted CCE experiments with six liquid crystal displays (LCDs) arranged to surround the observer (Fig. 1). As is typical of CCE experiments, there were repeated and novel layouts distributed across the six displays. We also used one layout that was memorized by the observer for 90 seconds before the experiment (explicit layout). This was to compare the effect of explicit knowledge with CCE. The target in the explicit layout was always on the 180° display.

### Experiment 1: Layouts in a 360-degree field

**Figure 2:**
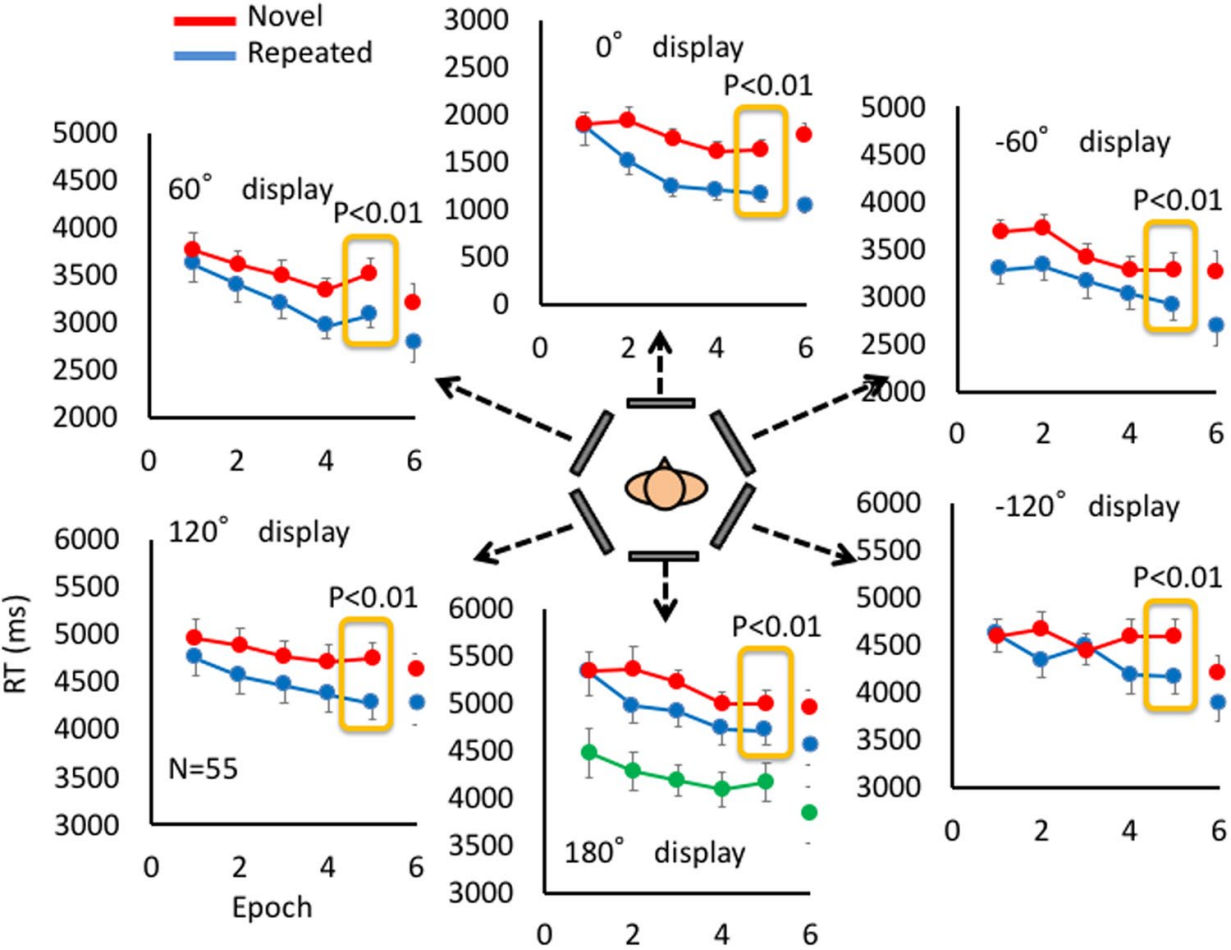
Reaction time to search for the target as a function of epoch (five blocks) for each display. The 0° display indicates the front display, the display in the front at the initial body location, and the other displays is labeled with the angle from the initial display with positive values in the counter-clockwise direction. The red line shows the RT of novel layouts and the blue one shows the RT of repeated layouts. The green line on 180° display shows the RT of explicit layout. The results of t-test result for the difference between repeated and novel layouts at the last epoch is shown for each display. The data points at epoch 6 (last five blocks) are the results of group A (26 observers), who completed 30 blocks.

The first experiment revealed the CCE for a spatial layout surrounding the observer. Figure 2 shows the changes in reaction time (RT) for correct responses as the experiment progressed (incorrect responses were 1.0% on average across observers). RT data at each epoch (comprising 4 blocks) are the average of 10 trials (two layouts of five blocks) for repeated and novel layouts and the average of five trials for the explicit layout. The number of trials, 8 per observer, for each epoch, was arbitrarily determined. Each panel shows the result for the target on each display. The arrangement of panels corresponds to the location of displays relative to the initial front display. A three-way ANOVA, two layout types (novel and repeated) x six displays x five epochs, was conducted, and the results showed significant main effects of all three factors (F(1,54) = 77.0, p < 0.001; F(5,270) = 144.3, p < 0.001 and F(4,216) = 22.3, p < 0.001 for layouts, display and epoch) and a significant interaction between layout and epoch (F(4,216) = 3.23, p = 0.018), illustrating the CCE in the present condition^30^. Because we are interested in the CCE for back displays, we performed a two-way ANOVA, two layout types (novel and repeated) x two epochs (first and last) for the RT averaged over three rear displays (±120° and 180°). The analysis showed significant main effects of layout type and epoch (F(1,54) = 17.4, p < 0.001 for layout and F(1,54) = 15.2, p < 0.001 for epoch) as well as a significant interaction between the layout type and epoch (F(1,54) = 6.99, p = 0.01). For each display, we used a one-tailed t-test to examine whether RT for repeated layouts was significantly shorter than that for novel layouts at the last epoch and found significant differences for all displays (t(55) = 3.18, p = 0.004; t(55) = 3.27, p = 0.003; t(55) = 3.40, p = 0.002; t(55) = 2.68, p = 0.013; t(55) = 3.71, p = 0.001; t(55) = 2.81, p = 0.009 for −120°, −60°, 0°, 60°,120°, 180°). Although testing the interaction between the layout type and epoch is usually required to demonstrate the learning effect, here we assumed no difference at the beginning of the experiment after the ANOVAs for all displays and three rear displays. This is because there could have been some learning effect even at the initial epoch after four trials, which makes it difficult to show the interaction in the present experiment. Reducing the number of blocks in an epoch is not a viable alternative because of the small number of trials in a block for each display.

Unawareness of the repeated use of layouts was confirmed by a recognition test, which asked the observer whether each repeated layout was the one presented during the search experiment among new layouts generated for the recognition test (see Methods). The recognition rate was 50.8% (±2.55% SE) and 57.8% (±3.11% SE) for the repeated and novel layouts, respectively. A t-test showed that it was not significantly different from 50% for repeated layouts (t(55) = 0.32, p = 0.75) while it was significant for novel layouts (t(25) = 2.54, p = 0.01). The learning effect of the explicit layout was found at earlier epochs. RT for the explicit layout was shorter than for novel ones, even at the first epoch (t(54) = 3.38, p < 0.001). The explicit layout was also tested in the recognition experiment, and the recognition rate was 74.5% (±5.93% SE), confirming explicit knowledge of the layout (the percentage was statistically larger than 50% (t(54) = 4.13, p < 0.001).

The chance-level performance for repeated layouts confirms that CCE is an implicit effect. On the other hand, it is puzzling that the correct rejection of novel layouts was better than chance. This does not support the use of explicit knowledge for repeated layouts because the chance-level performance for repeated layouts indicates that there was no available explicit knowledge during the recognition test. We confirmed that recognition performance was irrelevant to the CCE by showing the CCE for the data of observers with recognition rates lower than 50% (25 observers). A three-way ANOVA, two layout types (novel and repeated) x six displays x five epochs, showed significant main effects of all three factors (F(1,24) = 34.0, p < 0.001; F(5,120) = 72.2, p < 0.001 and F(4,96) = 8.6, p < 0.001 for layouts, display and epoch) and a significant interaction between layout and epoch (F(4,216) = 3.23, p = 0.018). A two-way ANOVA, two layout types (novel and repeated) x two epochs (first and last) for RT averaged over three rear displays (±120° and 180°) showed a significant effect of layout type (F(1,24) = 12.6, p = 0.002) as well as a significant interaction between layout type and the first and last epoch (F(1,24) = 6.43, p = 0.02), while it did not show a significant effect of epoch (F(1,24) = 3.44, p = 0.08).

Regarding correct rejection of novel layouts that was better than chance, we speculate that the explicit layout, which is not used in typical CCE experiments, may have caused higher rates of correct rejection for novel layouts in the recognition test. Recognition of the explicit layout was 75%, and the explicit layout must have provided familiarity. An impression of familiarity for some layouts may have biased the judgment of novel layouts to make them seem more novel due to lacking in familiarity. However, chance-level response with respect to repeated layouts is not consistent with this interpretation. If there was no difference between repeated and novel layouts with regard to recognition, both layout types should show fewer “yes” responses under the influence of explicit memory. The difference between repeated and novel layouts found here may provide an important property of layout representations learned implicitly, but we will leave this issue for future studies.

The CCE for the target in the displays including the ones located behind the observer suggests that the representation of the spatial layout surrounding the viewer is constructed through repeating visual search as in the case of layouts observed without changing the viewpoint. This can be attributed to the visual representation for full field scenes constructed of visual inputs from different visual directions with head and body movements. In such a representation, objects layout in the front display has a link to the target outside the visual field, such as the one on the 180° display. A critical question is whether the CCE found can be explained by only the layout representation of the display with the target, or whether the link to the target outside the visual field is required with a layout representation. Since there is a learning effect (CCE) for each display, the CCE within-display the target display may be sufficient to explain the CCE found with layouts surrounding the observer.

To answer the question, we divided the RT of each trial into two temporal periods. The first period is the time spent with the head orienting toward the target display, and the second period is the remaining time. The second period is the time to search for the target within the target display (within-display RT), and the first period is the time to search for the target display (across-display RT). Since the observer occasionally left the target display without target detection and came back again, the across-display RT includes the time spent on the other displays after leaving the first look at the target display, while the within-display RT is the total time spent on the target display.

We defined the time of gaze looking at the target display as the duration when the observer’s head was oriented toward a part of the display. Although we wished to use gaze data obtained by an eye tracker, we did not obtain sufficiently precise measurements of eye movements for many observers, and thus did not analyze eye movements. Division of RTs into two periods, within- and across-display RTs, allowed us to isolate CCE across displays from the CCE within display. This analysis was performed on data of 21 of the 26 observers for whom head movements were measured; data for 5 observers were excluded due to data loss for the last epoch.

**Figure 3:**
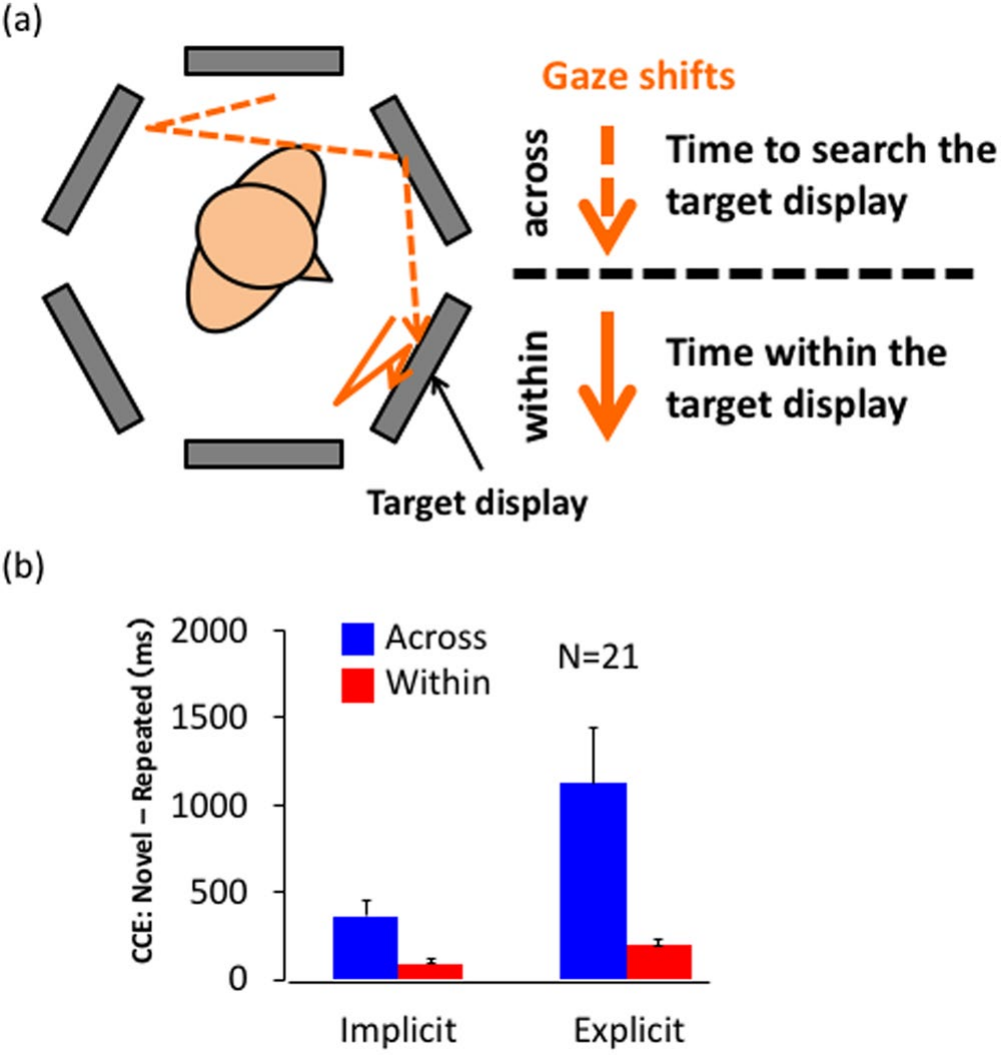
(**a**) Division of RT into within- and across-display periods. The within-display RT is the time to search for the target within the target display, and the across-display RT is the time to search for the target display. The time of first look of the target display was defined by the head orientation, the time when the head oriented to the target display. (**b**) CCE of within- and across-display RTs. The differences between novel and repeated layouts were calculated separately for within- and across-display RT. The RT results for the 0° display were not used.

From the within- and across-display RTs, the difference between repeated and novel layouts at the last epoch, i.e., the CCE, were calculated for all the display except for the front one, where little time for searching on the other displays was found. On average, 5.8% of total RT of the last block was spent on non-target displays for the implicit layouts and 9.6% for novel layouts when the target was on the front display, whereas the number was 83.1% and 80.9% when the target was presented on the other displays. The average within- and across-display CCEs for all displays except for the front one are shown in Fig. 3. A test showed that the both within- and across-display RTs for repeated layouts were significantly different from those for novel layouts (t(20) = 2.79 and p = 0.011 for within-display RT, and t(20) = 4.46, p < 0.001 for across-display RT). The results of the explicit layout showed larger within CCE, comparing with the implicit layout learning. Since the effect for across-display RT is based on global relationships between the scene and object, we call the effect contextual cueing effect for surrounding (CCES). The CCES supports the visual representation of the spatial layout surrounding the viewer, including outside the visual field. Since we could not use gaze data, the across-display and within RTs are approximation. Although a systematic bias between gaze and head orientations is known from eye-head coordination studies^31–35^, the same effect was expected for both the repeated and the novel layouts and the difference cannot be attributed to this effect. However, we conducted the next experiment to obtain a clearer support for the visual representation of surroundings.

### Experiment 2: Isolation of surroundings and local layouts

**Figure 4:**
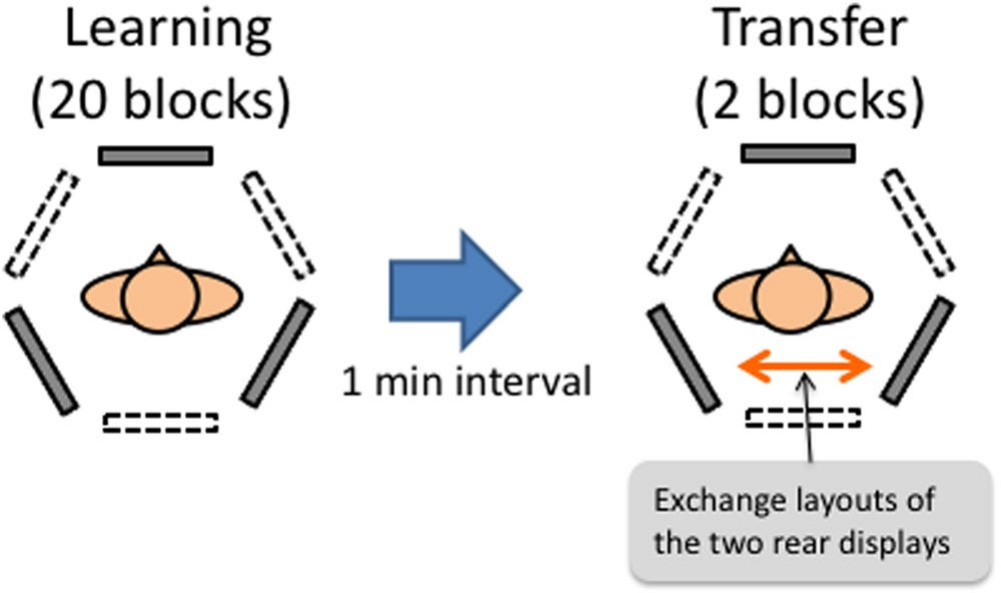
Experiment with three displays to examine the context effect of 0° display on the target location on ± 120° displays. After 20 blocks of visual search with three displays, layouts of the ± 120° were exchanged in transfer blocks. In the transfer blocks, RT should be lengthened if the observer tries to use the memorized relationship between the 0° display and the target on the ± 120° displays.

**Figure 5:**
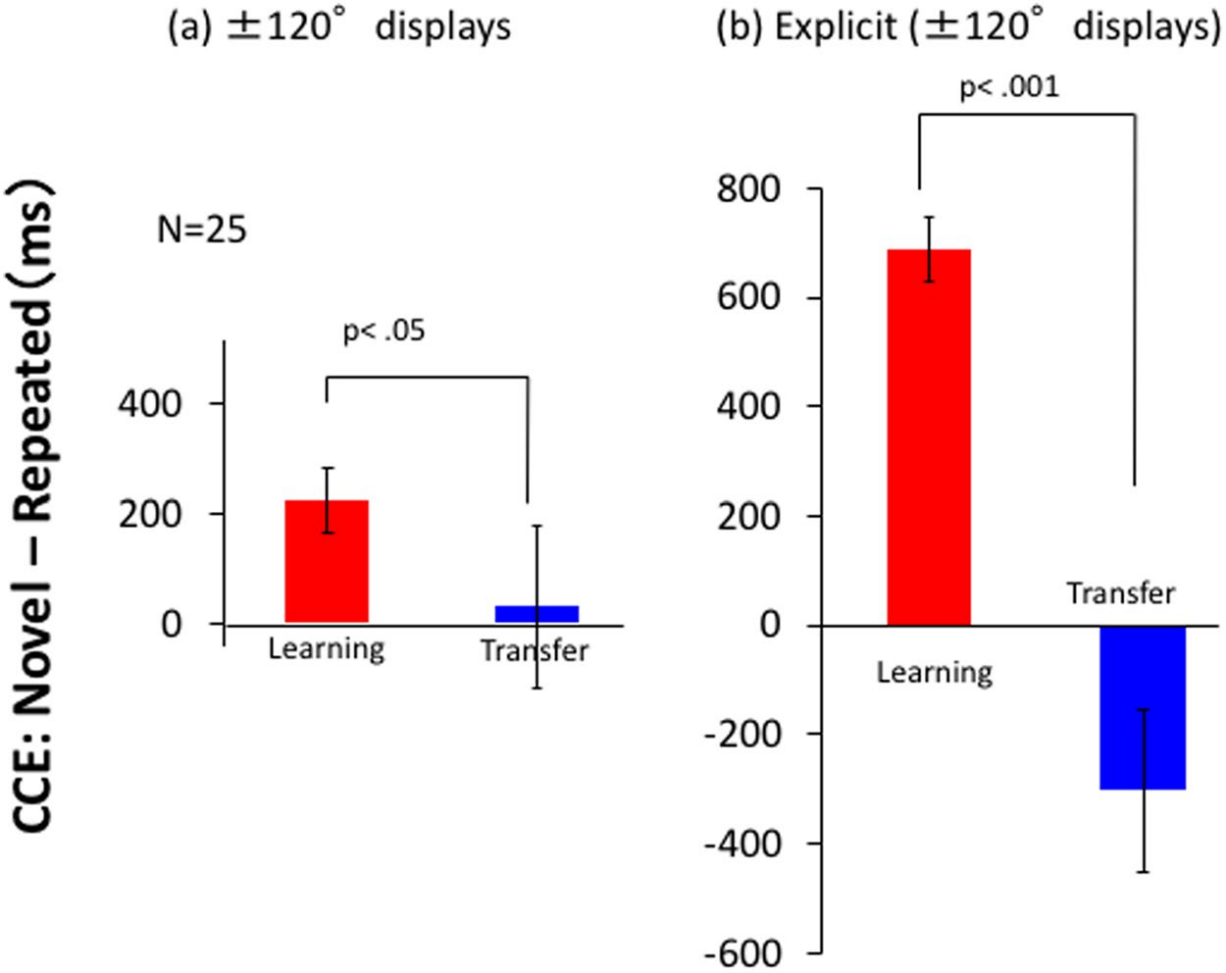
Comparison of CCE between the learning and transfer blocks for ± 120° displays (**a**) and for explicit layout. (**b**) The t-test result is shown for each comparison.

If the visual system integrates visual information seen from different viewpoints, which are not obtained simultaneously, for constructing spatial representations, the layout information on the display in front could provide information of the target located on a rear display in the experiment described above. We tested this assumption with three displays separated by 120° (Fig. 4), examining the cuing effect of the front layout on the target either on ± 120° displays. We repeated visual search experiment using the three displays. Then, we exchanged the layouts of the ± 120° displays to examine whether the CCE transfers to the same layout displayed on the other display (transfer phase) after 20 blocks of visual search (learning phase). Each block consisted of 12 repeated, 12 novel layouts and 1 explicit layout, the target of which was located on the 120° display for a half of the observers and on the −120° display for the other half. The learning effect of the relationship between the layout on the front display and the target location on either of the rear displays should be confused by the layout exchange in the transfer phase. Therefore, reduction of the CCE is expected if the relationship between the layout on the front display and target on a rear display has been learned during the learning phase. Figure 5(a) compares the differences between repeated and novel layouts for the last two blocks of the learning phase and the two blocks of the transfer phase (four trials for implicit and novel layouts and two trials for explicit layout if there was no incorrect response). Only RTs with correct responses were analyzed, and the proportion of incorrect responses was 0.9% on average. A clear reduction of the CCE was found for the transfer phase comparing with the learning phase. The difference was statistically significant (t(24) = 1.86, p = 0.038). Large reduction of CCE in the transfer phase was also found for explicit layouts (t(24) = 4.90, p < 0.001, which is about 800 ms and much larger than the effect for implicit layouts (Fig. 5(b)). This can be attributed to the strong negative effect due to the representations of the target location relative to the front layout obtained in the learning phase.

The recognition rate of this experiment was similar to that of Experiment 1. It was 51.0% (±8.16%) for the repeated layouts and the correct rejection rate was 63.7% (±3.19%) for novel layouts. The recognition rate for the repeated layout was not significantly different from 50% (t(25) = 0.28, p = 0.78) while the correct rejection rate for the novel layout was significantly different from 50% (t(25) = 4.29, p < 0.001). The recognition rate was 80.0% (±8.16%) for the explicit layout, which was statistically significantly different from 50% (t(25) = 3.67, p = 0.001).

## Discussion

Visual perception involves more than visual inputs, and the influence of the body and contextual information has been shown^4–7^. The present findings are related to functions of the mechanism for such general visual perception. First, PPS (peripersonal space) has been suggested to be specific to processing in the space near the body^8,9^. The representation of the surrounds investigated in the present study could be within the PPS, and therefore used for efficient action in the 3D world. Second, the current input from a certain retinal location is interpreted as a part of spatial and temporal continuity of the world. That is, visual input at a certain time and place is recognized as an object/event in the context of the scene or the environment surrounding the viewer^3–34^. The CCE of surrounds (CCES) supports that there is the contextual information in the representation built by multiple views across several seconds.

The CCES suggests that the visual system constructs representations for an area wider than the visible area, including the full field in the surrounding. While it may be difficult to image a full field representation visually, it is not difficult to do so auditorily. Audiovisual or multimodal representations should deal with space around the body, extending beyond the visual field. This is supported by a report of a common representation space for visual and auditory inputs^36^. Boundary extension error, where people remember seeing a wider image of a scene than was actually viewed^10,19^, is also found even when the input and test are in different modalities: either vision after touch or touch after vision (cross modal boundary extension)^37^. If a multimodal mechanism is associated with the CCES, a scene representation outside the visible area could be a natural consequence of sensory integration of multiple modalities.

The scene representations underlying the CCES should be constructed somewhere in the brain, and an important question is which brain site is responsible for the CCES. There are three factors related to this question: contextual cueing effect, location memory and boundary extension. First, several studies have shown that the CCE is related to the activity of the medial temporal lobe (MTL), including the hippocampus and retrosplenial cortex (RSC). A difference in brain activity in the MTL between novel and repeated layouts was reported in several fMRI studies^38–40^. While the MTL is often assumed to be related to only explicit memory^41^, recent studies of the CCE suggest a contribution of the MTL to implicit memory as well. The CCES found here may also be related to the same brain area. Second, place cells have been reported in the MTL^42,43^. Studies of place cells have suggested that spatial information independently of viewpoints is stored in the MTL, which could be the underlying mechanism of a cognitive map. Although place cells do not have to play a direct role for the CCES, there may be physiological units among many types of place cells in the MTL that are specialized to the CCES. Third, brain imaging studies have suggested that the retrosplenial cortex (RSC) is responsible for the boundary extension. Since the RSC is located close to the hippocampal spatial/memory system as well as visual areas, it may have a role in mediating between perception and memory for constructing spatial representations^44^. It is also known that the RSC contributes to context of scenes and navigation^17^. The CCE can be regarded as navigation effect of attention toward the target location by context or layout information. We do not consider the neural correlate for PPS here because most of the studies of neural responses related to PPS have focused not on the spatial representation but on modulation of action on visual sensitivity^13^.

These physiological studies mentioned above led us to speculate that the underlying mechanism of the CCES found here is in the MTL. The properties of MTL cells and/or activities are consistent with all aspects of the CCES. That is, learning effect of the context of scenes, expression of spatial layout including that outside the visual field, and navigation of attention to a target location. The MTL appears to be the most appropriate brain area for the CCES and investigation of its relationship to the CCES is an important issue in the future.

In conclusion, the present experiments revealed that the visual system constructs representations that link information within the visual field and information outside the visual field through repeated observation of the same spatial arrangements, that is, the CCES. The CCES is implicit and done without awareness of repeated observation of the same stimulus. Representations obtained by repetition without awareness are useful for moving around in familiar spaces and also in spaces that have structures in common with familiar places. Such representations should support actions in everyday life as well as specific actions for sports, driving, and so on.

## Method

### Observers

There were two experimental groups in Experiment 1: group A with 26 observers and group B with 29 observers. Group A had completed 30 blocks while group B completed 20 blocks. Since the number of blocks differed between the two groups (observers in group B were recruited to increase the number of observers with shorter experimental exposure), the first 20 blocks of group A data were used to compare the results between two types of layouts. The number of observers was 25 for Experiment 2. They had normal or corrected to normal vision (only contact lenses were allowed to avoid interference with the eye tracker). All observers were students of Tohoku University and were naïve to the purpose of the experiment. The experiments were approved by the Ethics Committee of Research Institute of Electrical Communication, Tohoku University, and the methods were carried out in accordance with the approved guidelines. All participants gave written informed consent.

### Stimuli

The observer’s task was to search for a target (“T”) among 35 distractors (“L”). The target and distractors were distributed among the six displays in random arrangements with the restriction that six items were presented in each display, which size was 60° × 45° when viewed from the center of the six displays. The locations of items were chosen randomly from intersections of an invisible 12 × 8 grid. The target “T” was rotated 90° to either the left or right, while distractor “Ls” were rotated by 0°, 90°, 180°, or 270°. The observer indicated the target direction (the heading direction of the end point of the vertical line of T) by pressing one of two buttons in the visual search experiments. The target and distractors were white (325.6 cd/m^2^) and were presented on a gray background (63.8 cd/m^2^). The target and distractors were 1.3° × 1.3° (Fig. 1(b)). Stimuli were generated using the Psychophysics Toolbox^45,46^ for MATLAB (MathWorks, U.S.A.).

There were two types of layouts as in a typical contextual cuing experiment. One was the repeated layout, which was used once in each block, and the other was a novel layout, which was used only once throughout a session. In addition, there was an explicit layout. The observer had 90 s to memorize the layout and the target location before the session. The explicit layout was used to compare the explicit and implicit learning processes. The layout indicates, here, the distribution of the items among all six displays. A repeated layout kept the locations of 36 items constant across all blocks with small jitters (±1.0° horizontally and ±1.5° vertically). There was no mixture of arrangements of some displays of a repeated layout with novel or other repeated layouts. All layouts consisted of a single target (“T”) and 35 distractors (“L”) distributed across six displays. Each block consisted of 25 trials: 12 repeated layouts, 12 novel layouts and 1 explicit layout. For repeated and novel layouts, there were two layouts with the target presented in each of the six displays while target location was in 180° display for the explicit layout.

Six items on a display used in the present study was sparser than that used in typical studies of contextual cueing (about 20 items). However, this was a condition where we expected to obtain the CCE. The CCE has been reported for layouts with set size of six^47^ or eight^30^, and the number of total items in the present experiments was within the range reported in typical CCE studies.

### Procedure – Experiment 1: layouts in a 360-degree field

The observer searched for a target of each repeated layout once in a block (30 or 20 times in total), through which the observer was expected to learn the layout. The observers stood at the center of six display and they moved the eye, head, body, and legs to turn a full 360 degrees to view all six displays. At the beginning of each trial, a fixation point was presented on the display, which was defined and used throughout the experiment as the initial front display. After a randomly chosen period between 0.5 and 1.5 s from a key press by the observer to initiate a trial, stimulus items were presented on the displays. The observer pressed either of two keys to indicate the target direction (the heading direction of the end point of the vertical line of T, either left or right, which judgement was easy for observers) when he/she found the target. The computer beeped following incorrect responses. The computer measured the reaction time (RT) for target detection. The presentation order of 25 layouts was randomized for each block. Before the experimental session, the observer performed one practice block with layouts that were not used in the experimental session.

The positions of eye and head orientations were recorded and analyzed after the experiments. Unfortunately, most of eye movement records were too noisy and unreliable perhaps because of the observers’ active movements during visual searches. Some of head movement data were also inappropriate for the analysis of RT division, and data of 5 of 26 observers for whom eye and head movements were recorded (group A) were excluded. The data were excluded when the number of trials without available head movement data was 2 or more of the 20 trials at the last epoch (two layout types x two layouts x five blocks).

### Procedure – Experiment 2: isolation of surroundings and local layouts

A session consisted transfer blocks in addition to learning blocks. The learning blocks were the same as in the first experiment except for the number of the displays and the block numbers. Only the front and ±120° displays were used and there were 20 blocks for learning. The number of items, therefore, was 18 in this experiment. With a 1-min interval after learning blocks, transfer blocks followed. In the transfer blocks, the layouts on the ±120° displays were exchanged without any change of the front display. The observer performed two transfer blocks. The different RT between the repeated and novel layouts, CCEs, averaged over last two learning blocks was compared to the same different RTs averaged over two transfer blocks (four trials of implicit and novel layouts, and two trials of explicit layout).

### Procedure – Recognition test

After visual search experiment, the observer performed a recognition test in both experiments to examine whether the observer had memory that was explicitly retrieved for each repeated layout both in Experiments 1 and 2. In the recognition test, a distractor replaced the target in each repeated layout. Thirteen repeated layouts (12 implicit and 1 explicit) from the search experiment were mixed with 12 novel layouts that had not been shown before. For each of the 25 layouts, the observer was asked whether he/she had seen the layout in the search blocks.

### Apparatus

The experiment was performed in a dark room using six liquid crystal displays (LCDs; MultiSync V321; NEC, Japan) with a refresh rate of 60 Hz, arranged in a hexagon^35^. The displays were centered at a height of 155 cm. An electromagnetic motion tracking system (FASTRAK; Polhemus, USA) was used to measure the orientation (azimuth, elevation) of two small, light-weighted sensors. One sensor was placed on the observer’s head, and the other was fixed on the observer’s back to record the orientation of the head and body in space at a 60 Hz sampling frequency with 4 ms latency and accuracy of 0.15°. Eye-in-head positions were recorded at 60 Hz by an eye tracker (EMR-9; NAC, Japan), which contains three cameras; two recorded the positions of the two eyes, and the scene camera in the middle had a field-of-view of 62° visual angle. The eye tracker has 71 ms delay and accuracy of 0.1° in measurements. All eye, head, and body orientation signals were synchronously recorded by a computer, which also controlled the stimulus displays. The eye tracker was used to measure eye orientation relative to head (gaze location on the image of the head camera). Combining head and eye orientations give the gaze location on a display^35^. The display was arranged 60 cm from the center of the area where the observer moved around during trials.
